# Enhanced lipid accumulation in microalgae through nanoparticle-mediated approach, for biodiesel production: A mini-review

**DOI:** 10.1016/j.heliyon.2021.e08057

**Published:** 2021-09-22

**Authors:** Rajesh Dev Sarkar, Hidam Bishworjit Singh, Mohan Chandra Kalita

**Affiliations:** Department of Biotechnology, Gauhati University, Guwahati, Assam, 781014, India

**Keywords:** Biodiesel, Nanoparticle, Biomolecules, Gene upregulation, Lipid production

## Abstract

Nanoparticle application in microalgae for enhanced lipid production is an ongoing work that leads towards the contribution in biodiesel production. During this decade, metal nanoparticles are constantly being reported to have numerous applications in diverse fields, because of their unique optical, electrical, and magnetic properties. They can interact with the biomolecules of cells and thereby alters cellular metabolisms, which in turn reflects their ability to regulate some primary or secondary metabolic pathways. Nanoparticles derived from metals like Fe, Cu, and Se are taking part in redox processes and their presence in many enzymes may modulate algal metabolisms. Besides by upregulating or downregulating the expression of several genes, nanoparticle exposure can alter gene expressions in many organisms. In microalgae such as *Chlorella vulgaris, C. pyrenoidosa, Scenedesmus obliquus, S. rubescens, Trachydiscus minutus, Parachlorella kessleri*, and *Tetraselmis suecica*; metal nanoparticle exposure in different environmental conditions have impacts on various physiological or molecular changes, thereby increasing the growth rate, biomass and lipid production. The present mini-review gives an insight into the various advantages and a future outlook on the application of nanoparticles in microalgae for biofuel production. Also, it can be proposed that nanoparticles could be useful in blocking or deactivating the AGPase enzyme (involved in the glucose to starch conversion pathway), binding to its active site, thereby increasing lipid production in microalgae that could be utilized for enhanced biodiesel production.

## Highlights

• Bioactive Nanoparticles modulate algal metabolism taking part in redox processes.
• Understanding NP-microalgae interaction will develop technology to enhance biofuel.
• Nanoparticles interacting with the enzyme AGPase may block its activity.
• *Chlorella vulgaris* has great potential in biofuel industry for high lipid content.
• Establishing biodiesel industries at rural sites will help unemployed fellows.

## Graphical abstract

**Figure:**
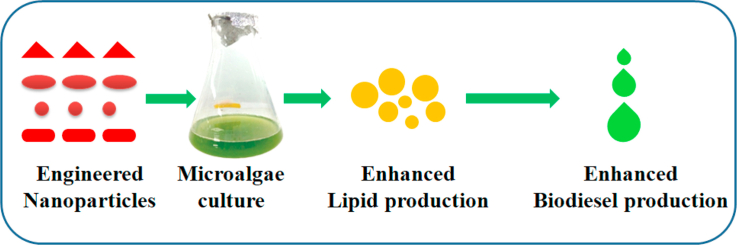

## 1 Introduction

Nanotechnology is currently the most flourishing sector in recent times due to its various applications and prospects. Nanoparticles (NPs) have been found in a varying range of applications in the past decade, and their unique optical, electrical and magnetic properties making them more penetrable, easy vectors and less toxic in nature; furthermore, a few NPs are also reported to be capable of activating water channels of cells (Eszenyi et al., 2011; Achuthan et al., 2014). Several NPs are already proved to interact with plant cells thereby increasing antioxidant enzyme activity (Khan, 2016; Latef et al., 2017) or by upregulating or downregulating specific genes (Almutairi, 2016; Wu et al., 2018). Having unique physical and chemical properties, the highly bioactive biogenic nanocrystallines viz., Fe, Mn, Zn, Cu, Co and Se due to their participation in different redox processes and their presence in many enzymes and complex proteins, may certainly modulate algal metabolism. Some studies have revealed that NP exposure alters the expression of the superoxide dismutase (SOD) gene along with other enzymes in plants (Mosa et al., 2018; Hossain et al., 2015; Alharby et al., 2016). Moreover, engineered NPs can also transport DNA and chemicals into an algal cell and it has been found that the size of NP is inversely proportional to the number of molecules present at the particle's surface (De Jong and Borm, 2008; Zhang et al., 2020b). The size, shape, surface area, and size distribution of NPs are the important deciding factors controlling their uptake by cells as it is highly dependent on cell wall pores. Nanotechnology has excellent potential in creating advanced systems for monitoring environmental conditions and improving the nutrient absorption of cells (Farooqui et al., 2016). Metal NP exposure in different microalgae (including *C. vulgaris*); might induce physiological, biochemical, or molecular changes thereby stimulating the growth, quality of biofuel or some defense mechanism may become active resulting in the formation of various pharmaceuticals, nutraceuticals such as pigments, exopolymers, peptides, and phytohormones. Moreover understanding the role of NPs in the alteration of conventional cellular processes of microalgae may develop technology to enhance both the quality and quantity of valuable cellular products including biofuel, pharmaceuticals and nutraceuticals. Few previous reports have showed that application of various NPs having a size range below 100 nm, can improve the growth and lipid production of a few microalgae as discussed in Table-1, however, the appropriate application of nanomaterials to facilitate algal cultures is still in its nascent phase and the mechanisms at large extent are not well understood. When fossil fuels have innumerable drawbacks and cause severe pollution and toxicity in the environment, biodiesel from algal lipid have been reported to be non-toxic, highly biodegradable, and contains no sulfur (Schenk et al., 2008). Several NPs can interact with different enzymes involved in the algal metabolic pathway thereby altering the normal metabolic pathway of cells (Phogat et al., 2018). The enzyme AGPase (ADP-glucose pyrophosphorylase) taking part in the starch biosynthesis process (Zabawinski et al., 2001), acts as a bottleneck in lipid production in microalgae. Hence blocking this starch synthesis pathway by inactivating AGPase enzyme (Li et al., 2010; Sharma et al., 2018) that catalyzes the conversion of glucose-1–phosphate to ADP-glucose; the precursor of starch biosynthesis, through the application of engineered NP could be an effective way to enhance lipid production in microalgae to a greater extent. This approach could be promising towards the enhancement of biodiesel production. Here in this present mini-review, the advantages of the application of engineered NPs in microalgae for enhanced lipid production and its future outlook has been discussed along with a proposed hypothesis on enhanced lipid production through blocking AGPase enzyme in microalgae.

## 2 Impacts of nanoparticles on microalgae species

Several NPs with specific features when applied in algal cultures, their impacts on algal cells have been reported to be associated with the growth and physiology; thereby increasing biomass, carbohydrate, or lipid content in the treated algal species.

From Table 1, it can be seen that the effects of NPs induce an increase in the total biomass, accumulation of carbohydrate and lipid content in *Chlorella, Scenedesmus, Tetraselmis,* and *Parachlorella* spp. Even though the overall effects of NP treatment in the above species of algae showed positive results but the difference in the accumulation of specific metabolites and their quantity may be due to differences in the physiology of the algae species. Among studied NPs, Fe based NPs have been most studied in the entire above species but, showed to be solely limited for only induction of the biomass with the highest being 51% in *P. kessleri* but these Fe based NPs are low inducers of lipids as compared to other NPs like Mg, Zn, and Pb that induces higher accumulation of lipids and the maximum effect being 393% in *C. vulgaris.* Besides, MgNP also induces a higher accumulation of carbohydrates in *C. vulgaris*. It can be inferred that FeNPs could be suitable for induction of biomass and other NPs like Mg, Zn, and Pb for induction of lipids and only MgNP for the induction of carbohydrates in algae in the future. A NP inducing higher accumulation of lipid might be helpful in the future for biofuel generation from algal biomass but further study is required to understand clearly since there is no clear evidence and enough data regarding the appropriate roles of size and concentration of different NPs.

**Table 1:** Impacts of several NPs on different Algal species.

NP (size in nm)	Conc. (mg/L)	Algae species	Impacts (% increments)			References
			Bio-mass	Carbo-hydrates	Lipids	
Fe (50)	100	*T. suecica*	-	-	41.9	Kadar et al. (2012)
Fe (-)	5.1	*T. minutus*	31.73	-	33.58	Pádrová et al. (2015)
Fe (-)	5.1	*P. kessleri*	51	-	14	Pádrová et al. (2015)
Fe_2_O_3_ (<30)	5	*S. obliquus*	-	-	39.6	He et al. (2017)
Fe_2_O_3_ (<50)	20–30	*C. pyrenoidosa*	33.75	-	15.29	Rana et al. (2020)
MgSO_4_ (100)	1000	*C. vulgaris*	-	-	118.23	Sarma et al. (2014)
Mg (82)	150–200	*C. vulgaris*	-	187.5	393.33	Sibi et al. (2017)
MgO (<50)	40	*S. obliquus*	-	-	18.5	He et al. (2017)
Cu (100)	0.67–4	*C. vulgaris*	20	-	-	Mykhaylenko and Zolotareva. (2017)
Cu (89)	10–20	*C. vulgaris*	-	87.5	86.67	Sibi et al. (2017)
Zn (92)	150	*C. vulgaris*	-	36.875	333.33	Sibi et al. (2017)
ZnO (50–70)	0.081	*S. rubescens*	-	-	27.27	Aravantinou et al. (2020)
Se (100)	0.4–4	*C. vulgaris*	40–45	-	-	Mykhaylenko and Zolotareva. (2017)
Pb (76)	50–100	*C. vulgaris*	-	56.25	206.67	Sibi et al. (2017)
C (<2)	5	*S. obliquus*	-	-	8.9	He et al. (2017)
SiC (25–100)	150	*Scenedesmus*. sp*.*	23.53	-	36.46	Ren et al. (2020)

NP (Nanoparticle); - (data not available); Conc. (Concentrations).

## 3 Technical feasibility of NP mediated biodiesel enhancement in microalgae

### 3.1 Feasibility of enhanced lipid production in microalgae

Having great propensity to interact with different biomolecules, NPs can control different cellular metabolisms. The application of nanomaterials extensively increases the microalgal growth and carbon di-oxide absorption (Vargas-Estrada et al., 2020). Several studies on the application of nanomaterials viz., Mg, Cu, Pb, Se, Fe, Zn, C and Si in certain microalgae such as *C. vulgaris*, *C. pyrenoidosa*, *S. obliquus*, *S. rubescens*, *T. minutus*, *P. kessleri*, and *T. suecica* revealed that NP application can enhance the microalgal growth, biomass production, carbohydrate and lipid contents (as documented in Table 1). This increase in the accumulation of lipids after application of the NPs could lead to an enhancement of biofuel production. Therefore, understanding the appropriate in-depth molecular mechanisms of NP-microalgae interaction leading towards the efficient enhancement of lipid production, will make the process of biofuel production feasible.

### 3.2 Large scale production of NPs and its cost

Since the synthesis of NP is becoming progressively straightforward, shortly large-scale production of NPs will be feasible at minimum cost and application of these engineered NPs for enhanced biodiesel production will be possible at a commercial scale (Chintagunta et al., 2021). Several microorganisms (bacteria, fungi) and plants extracts are already been identified which can easily synthesize NPs (through environment-friendly and inexpensive procedures) of desired characteristics with excellent activity in the biological field (Husen and Siddiqi, 2014; Sasidharan and Balakrishnaraja, 2014; Peralta-Videa et al., 2016; Fang et al., 2019). Soil bacteria viz., *Rhizobium* sp*.* (Jayavarthanan and Nanda, 2015), *Lactobacillus* sp. (Xu et al., 2018)*, Escherichia coli* (El-Shanshoury et al., 2011) have been reported to possess great potential for synthesizing NPs of desired structure at large scale and quickly at a reasonably low cost. However, the extraction of synthesized NPs from bacterial cells is still a tough challenge (Xu et al., 2018) since they mostly produce NPs in intracellular compartments. Though various techniques and protocols (Sonkusre et al., 2014; Forootanfara et al., 2014; Cruz et al., 2018) are described by a few Nanotechnologists but none of them are highly efficient for such a large-scale production and are highly expensive. Due to these limitations the process of extraction of NPs from bacterial cell are needed to be optimized aiming maximum yield of NPs at a minimum cost or there is a need of a bacterial species which can produce extracellular NPs at a large scale. On the other hand, the process of synthesis of NPs using plant extracts as reducing/capping agent don't have any complications, and it is faster than other processes as well as inexpensive (Saravanakumar et al., 2017; Zhang et al., 2020a; Kalita et al., 2021). During this decade the low-cost and commercial-scale production of NPs has been addressed by several researchers by adapting many other techniques (Schaidle et al., 2017) such as for the upscaled synthesis of CuNP, sputter deposition in ionic liquids was done (Meischein and Ludwig, 2021). In another work, to enhance the yield of AgNPs, synthesis was done by precipitation in a high-aqueous phase content reverse microemulsion (Sosa et al., 2010). Synthesis of Ce–Zn oxide NPs in continuous hydrothermal flow synthesis reactor has provided 17.5 times scale-up (on flow) over the existing laboratory-scale process (Tighe et al., 2013). In another case, a high throughput 16-channel millifluidic reactor has been developed that uses a multiphase gas-liquid flow to continuously produce colloidal Cesium Lead Bromide (CsPbBr_3_) quantum dots and it avoids the cost of a liquid carrier by using a gas carrier phase which is easier to separate and recycle (Wang et al., 2020). This is how with advancements in technology for bioactive NP synthesis, the availability and cost of NPs will certainly go down very soon in near future. Hence more extensive research is required in technology advancement for large scale production of NPs with specific features at very low-cost for application in microalgae culture for the purpose of enhanced biofuel production.

### 3.3 Biofuel industry growth

With the growing demand for cheaper, reliable, and sustainable energy sources to alleviate acute vulnerability to the fossil fuel supply chain and reach the increasing fuel demand, it is predicted to possess a positive impact on the microalgal biofuel industry growth. This algal biofuel industry has three main problems-maintaining algae culture across various climates, high water demand, and lack of technology for commercialization. As a result, the market is predicted to foresee regional partnerships and collaborations to maximize the assembly and technology exchange for giant scale productions (Grand View Research Report, 2017). It seems probable that global climate change, greenhouse emission effects, depleting freshwater resources in some regions, growth in human population, and shortages of agricultural land will favour the use of third-generation biofuel production system such as microalgae and Sarpal et al. (2016) revealed that *Chlorella* sp. oil is promising feedstocks for biodiesel. Though maintaining axenic culture is a challenge with raceway ponds (Bell et al., 2016) for biomass production, it can be minimized by regular cleaning and taking proper care of the culture. Producion of approximately 5,000–15,000 gal of biodiesel is possible from microalgae per acre per year (Spolaore et al., 2006; Chisti, 2007; Behera et al., 2015), but yet this is insufficient for replacement of fossil fuel, hence application of NPs, shifting the biodiesel production to a greater extent from different highly efficient microalgal species, are going to be more beneficial. Besides the production of primary product (biodiesel), several valuable co-products such as algal meal, omega-3 fatty acids, glycerin from these biorefineries may also improve the economics of the entire system (Peters et al., 2019) thereby contributing to the industry growth.

### 3.4 Advantage of NP application over the transgenic approach

Though different genetic engineering strategies applied in *Chlorella* sp. for biodiesel improvement such as regulon engineering (manipulation of a regulatory gene or transcription factor that regulates a group of gene), optimizing light harvesting efficiency, enhancing carbon capture, manipulating precursor building pathways, blocking starch synthesis, modulating fatty acid synthesis, blocking lipolysis, stimulating TAG (triacylglycerol) synthesis, overexpression of acetyltransferases, manipulation of Calvin cycle are in progress but the impact on human health and environmental risks are the major concerns with transgenic microalgae if exposed to natural ecosystems (Sharma et al., 2018) as they are the primary producers in aquatic ecosystems and may cause catastrophe in the ecosystem. Although a study proposed that the risk of genetically modified algae could be reduced by coupling the expression of both, the transgene of interest and antisense or RNAi of a gene that increases robustness in natural systems (Gressel et al., 2013) but this approach might induce the excess transgene-load in the genetically modified algae. Moreover, the stability of transgene in genetically modified microalgae is also a major concern, and due to the burden of excess transgene and specific nutritional need, they are also mostly unable to adapt well to natural systems (Kumar, 2015). A case study has reported that the transgenic microalgae *Acutodesmus dimorphus* could not survive in natural pond in presence of other native strains of algae (Szyjka et al., 2017). In addition to this, the disposal of residual substances of transgenic algae is also a major concern for the environment (Abdullah et al., 2019) which reflects that the engineered transgenic algae may not be an efficient strategy for enhanced biofuel production; however NP-mediated enhancement of biodiesel production will have an advantage over all these strategies.

### 3.5 NP-enzyme interaction for enhanced biofuel production

**Figure 1:**
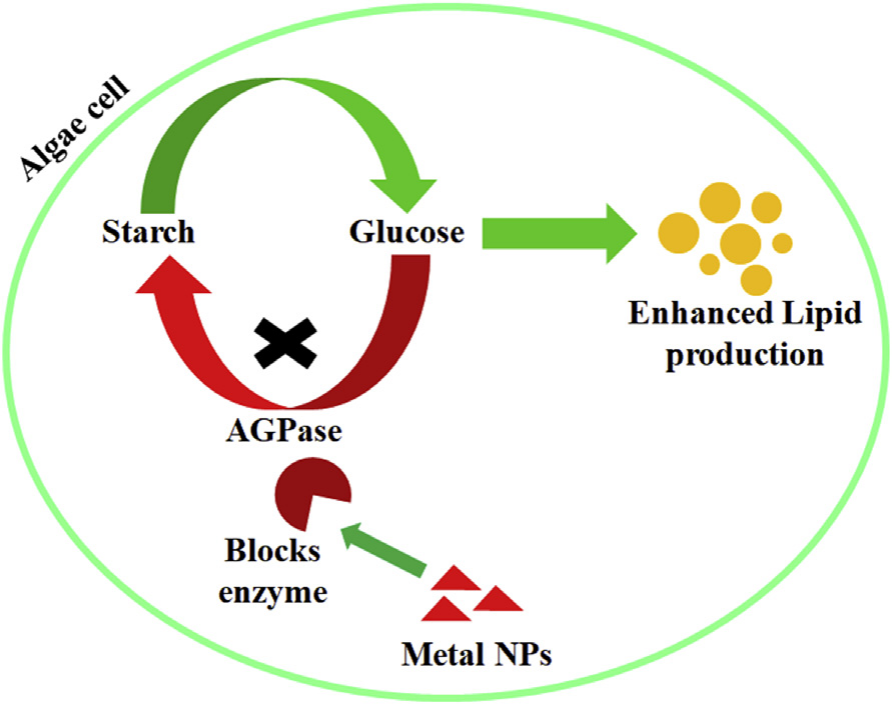
A hypothetical model on blocking AGPase by engineered NPs to block Starch Synthesis pathway. (Engineered NPs with specific features may bind the active site of AGPase, thereby blocking its activity, and hence blocking of glucose to starch synthesis pathway may occur. This is how the glucose molecules are expected to undergo lipid synthesis, which will enhance biodiesel production).

NPs can alter cellular metabolic pathways by interacting with biomolecules including enzymes (Phogat et al., 2018; Baimanov et al., 2019), besides, immobilization of enzymes are also possible by nanomaterials (Chen et al., 2017). To control certain enzyme functions, NPs can be selected to interact with those enzymes specifically and their interactions are mainly dependent upon the specific features of NPs including size, shape, surface properties and the oxidation state of the NPs (Wu et al., 2009). NPs can selectively inhibit enzyme activities by competitive inhibition, non-competitive inhibition or denaturation (Kopp et al., 2017). Therefore, fabrication of such NPs with specific features to bind certain enzymes or proteins could make it possible to regulate their activity. Further, it has also been reported by a research group that ZnO NPs with shapes pyramids (base 15 nm, side edges 18 nm), plates (diameter 18.4 nm), and sphere (diameter 4.4 nm) having electrokinetic zeta potential of +30.8, +27.6 and +33.7 mV respectively, successfully inhibited the activity of β-galactosidase (GAL) enzyme of *Escherichia coli* (Cha et al., 2015). SiO_2_ NPs with diameter 4, 20 and 100 nm and surface area per particle 50, 1257 and 31416 nm^2^ respectively, when bind with the enzyme lysozyme in its surface, provoke structural changes in the enzyme resulting in the reduction of enzyme activity in a size-dependent manner (Vertegel et al., 2004). Functionalizing NPs by modifying its large surface area with organic molecules through covalent or non-covalent interactions has been discussed to prevent non-specific bindings and helps in recognizing specific enzymes or biomolecules (Wu et al., 2009). In this circumstance, an extensive research work is necessary to document and establish the molecular mechanism of binding of a particular NP (having specific features) with AGPase enzyme (Figure 1), so that it could be applicable in blocking or inhibiting its activity to inhibit starch synthesis pathway. This approach in turn will help in overcoming the bottleneck of lipid production in microalgae thereby contributing in enhanced biofuel production.

## 4 Challenges in the enhancement of biodiesel production from microalgae by NP mediated strategy

Though a high-quality biofuel generated from several microalgae, the production rate is insufficient to replace fossil fuel; here in this circumstance, nanotechnology is expected to shift the biofuel production rate to a greater extent. Few evidences are available where the application of several NPs at certain concentrations improved the quantity of lipid content extensively in several microalgae (as discussed in Table 1) however, the appropriate application of nanomaterials to facilitate algal cultures is still in its primitive phase and the molecular mechanisms at large extents are not well understood. Targeting and blocking of starch synthesis pathway by NP application will also need an extensive research (Rawat et al., 2018). Many metal NPs have great bioactivity (Rai et al., 2015), but which one has the best activity (at minimum cost) and targets which metabolic pathways of microalgae such as *C. vulgaris, C. pyrenoidosa*, *S. obliquus*, *S. rubescens*, *T. minutus*, *P. kessleri*; under normal growth conditions are needed to be determined by extensive research. Currently maintaining axenic culture in raceway ponds, easy and low-cost processing of biofuel and maintaining high quality are also big challenges in this field. Many bacteria have great potential in synthesizing bioactive NPs of desired quality on large scale (Sasidharan and Balakrishnaraja, 2014) however; extraction of NPs from the bacteria is a tough challenge which is required to be optimized aiming maximum yield of NPs at a minimum cost, and at the same time conserving the bioactivity of the NPs. A minor drawback regarding algal biofuel is that they are less stable because of their highly unsaturated and volatile nature, and thus more prone to degradation however this problem has been addressed in some research works by generating potential solutions (Rawat et al., 2018).

## 5 Social implications of algal biodiesel production

Since the algal culture doesn't require much expertise hands in this field, establishing biodiesel industries at rural sites could help unemployed fellows to earn their livelihood by cultivating algae at the domestic level. Moreover, these microalgae can be grown on non-arable land and saline water which is not suitable for other purposes (Batterton and Van Baalen, 1971). Besides, the waste algal biomass generated after isolating the biodiesel, can be used to feed fishes. Since the algal growth rate is so slow in comparison to the demands for biodiesel production, hence the use of algae in biodiesel production has become inefficient, but once the concept of application of nanotechnology in algal culture is properly established, it will shift the growth rate and biodiesel production to a greater extent so as to use as a potent alternative to fossil diesel fuel. Since biodiesel is a source of green energy, its harmful effects in the environment will be minimum (Chauhan and Shukla, 2011). Microalgae are the fastest growing photosynthetic organisms, requiring 1.8 tons of CO_2_ for the production of 1 ton of algal biomass (Sudhakar et al., 2011), thereby reducing the main factor of climate change. Being a non-renewable source, fossil fuels are depleting rapidly and expected to be diminished by the middle of this century, besides they are directly related to air, water and soil pollution. In these drastic situations, biofuel from renewable sources can be a potential alternative towards reducing our dependency on fossil fuel and assist to maintain a healthy global environment and economic sustainability.

## 6 Conclusion

Microalgae have been used as feedstock for the production of biofuel since they have many advantages like fast growth, higher lipid accumulation and photosynthetic yield at low cost. Microalgae-NP interaction provides an opportunity for enhancement of lipid production in an extensive proportion that could lead towards significant contribution in biofuel production. In health or agriculture sector, NP applications are already proved to be affordable and during this decade constant work is going on in technology advancement for low-cost and large-scale production of NPs, which reflects the probability of its application in biofuel production soon in near future. Even though the exact mechanisms of the activity of NPs in biological field is not appropriately known, however engineered NPs with specific characteristics have been proved to have the ability to selectively block or inhibit the activity of certain enzymes. Applications of engineered NPs on microalgal culture to selectively induce the inhibition or inactivity of the AGPase enzyme that is involved in the starch biosynthesis pathway, may lead to the diversion of the metabolic intermediates towards the enhanced biosynthesis of lipids. Understanding the NP-microalgae interaction mechanisms at the molecular level will strengthen our current hypothesis and its promising applications ensuring our control on algal biodiesel production. Future investigations should target understanding the in-depth molecular mechanisms behind the enhancement of lipid production in microalgae through NP application.

## Declarations

### Author contribution statement

All authors listed have significantly contributed to the development and the writing of this article.

### Funding statement

This work was supported by the 10.13039/501100001409DST (Govt. of India) through the DST-INSPIRE Fellowship grant.

### Data availability statement

No data was used for the research described in the article.

### Declaration of interests statement

The authors declare no conflict of interest.

### Additional information

No additional information is available for this paper.

## References

[bib1] Abdullah B., Muhammad S.A.F.A.S., Shokravi Z., Ismail S., Kassim K.A., Mahmood A.N., Aziz M.M.A. (2019). Fourth generation biofuel: a review on risks and mitigation strategies. Renew. Sustain. Energy Rev..

[bib2] Achuthan T., Sasidharan S., Balakrishnaraja R. (2014). Utilization of organic selenium nanoparticles to inhibit algal growth. Nat. Environ. Pollut. Technol..

[bib3] Alharby H.F., Metwali E.M., Fuller M.P., Aldhebiani A.Y. (2016). The alteration of mRNA expression of SOD and GPX genes, and proteins in tomato (Lycopersicon esculentum Mill) under stress of NaCl and/or ZnO nanoparticles. Saudi J. Biol. Sci..

[bib4] Almutairi Z.M. (2016). Influence of silver nano-particles on the salt resistance of tomato (Solanum lycopersicum) during germination. Int. J. Agric. Biol..

[bib5] Aravantinou A.F., Andreou F., Manariotis I.D. (2020). Long-term toxicity of ZnO nanoparticles on Scenedesmus rubescens cultivated in semi-batch mode. Nanomaterials.

[bib6] Baimanov D., Cai R., Chen C. (2019). Understanding the chemical nature of nanoparticle–protein interactions. Bioconjugate Chem..

[bib7] Batterton J.C., Van Baalen C. (1971). Growth responses of blue-green algae to sodium chloride concentration. Arch. Mikrobiol..

[bib8] Behera S., Singh R., Arora R., Sharma N.K., Shukla M., Kumar S. (2015). Scope of algae as third generation biofuels. Front. Bioengin. Biotechnol..

[bib9] Bell T.A., Prithiviraj B., Wahlen B.D., Fields M.W., Peyton B.M. (2016). A lipid-accumulating alga maintains growth in outdoor, alkaliphilic raceway pond with mixed microbial communities. Front. Microbiol..

[bib10] Cha S.H., Hong J., McGuffie M., Yeom B., VanEpps J.S., Kotov N.A. (2015). Shape-dependent biomimetic inhibition of enzyme by nanoparticles and their antibacterial activity. ACS Nano.

[bib11] Chauhan S.K., Shukla A. (2011).

[bib12] Chen M., Zeng G., Xu P., Lai C., Tang L. (2017). How do enzymes ‘meet’nanoparticles and nanomaterials?. Trends Biochem. Sci..

[bib13] Chintagunta A.D., Kumar A., Jeevan Kumar S.P., Verma M.L., Rajendran S., Qin J., Gracia F., Lichtfouse E. (2021). Metal and Metal Oxides for Energy and Electronics.

[bib14] Chisti Y. (2007). Biodiesel from microalgae. Biotechnol. Adv..

[bib15] Cruz D.M., Mi G., Webster T.J. (2018). Synthesis and characterization of biogenic selenium nanoparticles with antimicrobial properties made by *Staphylococcus aureus*, methicillin-resistant *Staphylococcus aureus* (MRSA), *Escherichia coli*, and *Pseudomonas aeruginosa*. J. Biomed. Mater. Res..

[bib16] De Jong W.H., Borm P.J. (2008). Drug delivery and nanoparticles: applications and hazards. Int. J. Nanomed..

[bib17] El-Shanshoury A.E.R.R., ElSilk S.E., Ebeid M.E. (2011). Extracellular biosynthesis of silver nanoparticles using Escherichia coli ATCC 8739, Bacillus subtilis ATCC 6633, and Streptococcus thermophilus ESh1 and their antimicrobial activities. ISRN Nanotechnology.

[bib18] Eszenyi P., Sztrik A., Babka B., Prokisch J. (2011). Elemental, nano-sized (100-500 nm) selenium production by probiotic lactic acid bacteria. International Journal of Bioscience, Biochemistry and Bioinformatics.

[bib19] Fang X., Wang Y., Wang Z., Jiang Z., Dong M. (2019). Microorganism assisted synthesized nanoparticles for catalytic applications. Energies.

[bib20] Farooqui A.R.E.E.B.A., Tabassum H.E.E.N.A., Ahmad A.S.A.D., Mabood A.B.D.U.L., Ahmad A.D.N.A.N., Ahmad I.Z. (2016). Role of nanoparticles in growth and development of plants: a review. Int. J. Pharma Bio Sci..

[bib21] Forootanfara H., Zare B., Fasihi-Bam H., Amirpour-Rostami S., Ameri A., Shakibaie M., Nami M.T. (2014). Biosynthesis and characterization of selenium nanoparticles produced by terrestrial actinomycete *Streptomyces microflavus* strain FSHJ31. Res. Rev.: J. Microbiol. Biotechnol..

[bib22] Grand View Research (2017). http://www.prnewswire.com/news-releases/algae-biofuel-market-worth-1073-billion-by-2025&ndash;growth-rate-88-grand-view-research-inc-616586654.html.

[bib23] Gressel J., van der Vlugt C.J., Bergmans H.E. (2013). Environmental risks of large scale cultivation of microalgae: mitigation of spills. Algal Res..

[bib24] He M., Yan Y., Pei F., Wu M., Gebreluel T., Zou S., Wang C. (2017). Improvement on lipid production by Scenedesmus obliquus triggered by low dose exposure to nanoparticles. Sci. Rep..

[bib25] Hossain Z., Mustafa G., Komatsu S. (2015). Plant responses to nanoparticle stress. Int. J. Mol. Sci..

[bib26] Husen A., Siddiqi K.S. (2014). Plants and microbes assisted selenium nanoparticles: characterization and application. J. Nanobiotechnol..

[bib27] Jayavarthanan R., Nanda A. (2015). Antibiogram of silver nanoparticles synthesized from Rhizobium species. Der Pharm. Lett..

[bib28] Kadar E., Rooks P., Lakey C., White D.A. (2012). The effect of engineered iron nanoparticles on growth and metabolic status of marine microalgae cultures. Sci. Total Environ..

[bib29] Kalita C., Sarkar R.D., Verma V., Bharadwaj S.K., Kalita M.C., Boruah P.K., Das M.R., Saikia P. (2021). Bayesian modeling coherenced green synthesis of NiO nanoparticles using camellia sinensis for efficient antimicrobial activity. BioNanoScience.

[bib30] Khan M.N. (2016). Nano-titanium dioxide (nano-TiO2) mitigates NaCl stress by enhancing antioxidative enzymes and accumulation of compatible solutes in tomato (Lycopersicon esculentum Mill.). J. Plant Sci..

[bib31] Kopp M., Kollenda S., Epple M. (2017). Nanoparticle–protein interactions: therapeutic approaches and supramolecular chemistry. Acc. Chem. Res..

[bib32] Kumar S. (2015). GM algae for biofuel production: biosafety and risk assessment. Collect Biosaf Rev.

[bib33] Latef A.A.H.A., Alhmad M.F.A., Abdelfattah K.E. (2017). The possible roles of priming with ZnO nanoparticles in mitigation of salinity stress in lupine (Lupinus termis) plants. J. Plant Growth Regul..

[bib34] Li Y., Han D., Hu G., Dauvillee D., Sommerfeld M., Ball S., Hu Q. (2010). Chlamydomonas starchless mutant defective in ADP-glucose pyrophosphorylase hyper-accumulates triacylglycerol. Metab. Eng..

[bib35] Meischein M., Ludwig A. (2021). Upscaling nanoparticle synthesis by sputter deposition in ionic liquids. J. Nanoparticle Res..

[bib36] Mosa K.A., El-Naggar M., Ramamoorthy K., Alawadhi H., Elnaggar A., Wartanian S., Ibrahim E., Hani H. (2018). Copper nanoparticles induced genotoxicty, oxidative stress, and changes in Superoxide Dismutase (SOD) gene expression in cucumber (Cucumis sativus) plants. Front. Plant Sci..

[bib37] Mykhaylenko N.F., Zolotareva E.K. (2017). The effect of copper and selenium nanocarboxylates on biomass accumulation and photosynthetic energy transduction efficiency of the green algae *Chlorella vulgaris*. Nanoscale research letters.

[bib38] Pádrová K., Lukavský J., Nedbalová L., Čejková A., Cajthaml T., Sigler K., Vítová M., Řezanka T. (2015). Trace concentrations of iron nanoparticles cause overproduction of biomass and lipids during cultivation of cyanobacteria and microalgae. J. Appl. Phycol..

[bib39] Peralta-Videa J.R., Huang Y., Parsons J.G., Zhao L., Lopez-Moreno L., Hernandez-Viezcas J.A., Gardea-Torresdey J.L. (2016). Plant-based green synthesis of metallic nanoparticles: scientific curiosity or a realistic alternative to chemical synthesis?. Nanotechnol. Environ. Engin..

[bib40] Peters M., Stokes J., Tu R. (2019).

[bib41] Phogat N., Kohl M., Uddin I., Jahan A. (2018). Precision Medicine.

[bib42] Rai M., Ingle A.P., Gupta I., Brandelli A. (2015). Bioactivity of noble metal nanoparticles decorated with biopolymers and their application in drug delivery. Int. J. Pharm..

[bib43] Rana M.S., Bhushan S., Sudhakar D.R., Prajapati S.K. (2020). Effect of iron oxide nanoparticles on growth and biofuel potential of Chlorella spp. Algal Research.

[bib44] Rawat D.S., Joshi G., Pandey J.K., Lamba B.Y., Kumar P. (2018). Algal biodiesel stabilization with lower concentration of 1: 3 ratios of binary antioxidants–Key factors to achieve the best synergy for maximum stabilization. Fuel.

[bib45] Ren H.Y., Dai Y.Q., Kong F., Xing D., Zhao L., Ren N.Q., Ma J., Liu B.F. (2020). Enhanced microalgal growth and lipid accumulation by addition of different nanoparticles under xenon lamp illumination. Bioresour. Technol..

[bib46] Saravanakumar A., Peng M.M., Ganesh M., Jayaprakash J., Mohankumar M., Jang H.T. (2017). Low-cost and eco-friendly green synthesis of silver nanoparticles using Prunus japonica (Rosaceae) leaf extract and their antibacterial, antioxidant properties. Artificial Cells, Nanomed. Biotechnol..

[bib47] Sarma S.J., Das R.K., Brar S.K., Le Bihan Y., Buelna G., Verma M., Soccol C.R. (2014). Application of magnesium sulfate and its nanoparticles for enhanced lipid production by mixotrophic cultivation of algae using biodiesel waste. Energy.

[bib48] Sarpal A.S., Costa I.C., Teixeira C.M.L.L., Filocomo D., Candido R., Silva P.R., Cunha V.S., Daroda R.J. (2016). Investigation of biodiesel potential of biomasses of microalgaes chlorella, spirulina and tetraselmis by NMR and GC-MS techniques. J. Biotechnol. Biomater..

[bib49] Sasidharan S., Balakrishnaraja R. (2014). Comparison studies on the synthesis of selenium nanoparticles by various microorganisms. Int J Pure App Biosci.

[bib50] Schaidle J.A., Habas S.E., Baddour F.G., Farberow C.A., Ruddy D.A., Hensley J.E., Brutchey R.L., Malmstadt N., Robota H. (2017). Catalysis.

[bib51] Schenk P.M., Thomas-Hall S.R., Stephens E., Marx U.C., Mussgnug J.H., Posten C., Kruse O., Hankamer B. (2008). Second generation biofuels: high-efficiency microalgae for biodiesel production. Bioenergy Res..

[bib52] Sharma P.K., Saharia M., Srivstava R., Kumar S., Sahoo L. (2018). Tailoring microalgae for efficient biofuel production. Front. Marine Sci..

[bib53] Sibi G., Ananda Kumar D., Gopal T., Harinath K., Banupriya S., Chaitra S. (2017). Metal nanoparticle triggered growth and lipid production in Chlorella vulgaris. Int J Scientific Res Environ Sci Toxicol.

[bib54] Sonkusre P., Nanduri R., Gupta P., Cameotra S.S. (2014). Improved extraction of intracellular biogenic selenium nanoparticles and their specificity for cancer chemoprevention. J. Nanomed. Nanotechnol..

[bib55] Sosa Y.D., Rabelero M., Treviño M.E., Saade H., López R.G. (2010). High-yield synthesis of silver nanoparticles by precipitation in a high-aqueous phase content reverse microemulsion. J. Nanomater..

[bib56] Spolaore P., Joannis-Cassan C., Duran E., Isambert A. (2006). Commercial applications of microalgae. J. Biosci. Bioeng..

[bib57] Sudhakar K., Suresh S., Premalatha M. (2011). An overview of CO_2_ mitigation using algae cultivation technology. Int. J. Chem. Res..

[bib58] Szyjka S.J., Mandal S., Schoepp N.G., Tyler B.M., Yohn C.B., Poon Y.S., Villareal S., Burkart M.D., Shurin J.B., Mayfield S.P. (2017). Evaluation of phenotype stability and ecological risk of a genetically engineered alga in open pond production. Algal Research.

[bib59] Tighe C.J., Cabrera R.Q., Gruar R.I., Darr J.A. (2013). Scale up production of nanoparticles: continuous supercritical water synthesis of Ce–Zn oxides. Ind. Eng. Chem. Res..

[bib60] Vargas-Estrada L., Torres-Arellano S., Longoria A., Arias D.M., Okoye P.U., Sebastian P.J. (2020). Role of nanoparticles on microalgal cultivation: a review. Fuel.

[bib61] Vertegel A.A., Siegel R.W., Dordick J.S. (2004). Silica nanoparticle size influences the structure and enzymatic activity of adsorbed lysozyme. Langmuir.

[bib62] Wang L., Karadaghi L.R., Brutchey R.L., Malmstadt N. (2020). Self-optimizing parallel millifluidic reactor for scaling nanoparticle synthesis. Chem. Commun..

[bib63] Wu H., Shabala L., Shabala S., Giraldo J.P. (2018). Hydroxyl radical scavenging by cerium oxide nanoparticles improves Arabidopsis salinity tolerance by enhancing leaf mesophyll potassium retention. Environ. Sci.: Nano.

[bib64] Wu Z., Zhang B., Yan B. (2009). Regulation of enzyme activity through interactions with nanoparticles. Int. J. Mol. Sci..

[bib65] Xu C., Guo Y., Qiao L., Ma L., Cheng Y., Roman A. (2018). Biogenic synthesis of novel functionalized selenium nanoparticles by Lactobacillus casei ATCC 393 and its protective effects on intestinal barrier dysfunction caused by enterotoxigenic Escherichia coli K88. Front. Microbiol..

[bib66] Zabawinski C., Van Den Koornhuyse N., d'Hulst C., Schlichting R., Giersch C., Delrue B., Lacroix J.M., Preiss J., Ball S. (2001). Starchless mutants of Chlamydomonas reinhardtii lack the small subunit of a heterotetrameric ADP-glucose pyrophosphorylase. J. Bacteriol..

[bib67] Zhang D., Ma X.L., Gu Y., Huang H., Zhang G.W. (2020). Green synthesis of metallic nanoparticles and their potential applications to treat cancer. Front. Chem..

[bib68] Zhang Y., Zhang Y., Ma C., Fu G., Mu S., Liu X., Zhang H. (2020).

